# Risk factors and Compliance of surviving sepsis campaign: A retrospective cohort study at tertiary care hospital

**DOI:** 10.12669/pjms.38.1.3992

**Published:** 2022

**Authors:** Farheen Yousuf, Ayesha Malik, Ayesha Saba, Sana Sheikh

**Affiliations:** 1 Dr. Farheen Yousuf, FRCOG, MCPS(HPE), FCPS. Department of Obstetrics and Gynecology, Aga Khan University, Karachi, Pakistan; 2 Dr. Ayesha Malik, MRCOG, FCPS. Department of Obstetrics and Gynecology, Aga Khan University, Karachi, Pakistan; 3 Dr. Ayesha Saba, FCPS. Department of Obstetrics and Gynecology, Aga Khan University, Karachi, Pakistan; 4 Dr. Sana Sheikh, Masters in Epidemiology and Biostatics Department of Obstetrics and Gynecology, Aga Khan University, Karachi, Pakistan

**Keywords:** Sepsis, Surviving sepsis campaign, Puerperal pyrexia, Maternal mortality

## Abstract

**Background and Objective:**

Sepsis is one of the leading causes of direct maternal mortality in Pakistan. It is recommended that the first three hours after the presentation are crucial. During this time implementation of surviving sepsis campaign resuscitation bundles reduces maternal mortality. Our objective was to assess the factors contributing to puerperal sepsis and the compliance of “surviving sepsis campaign resuscitation bundles in puerperal sepsis” for the management of puerperal sepsis.

**Methods:**

This was a retrospective record review for five years from January 2011-December 2015. All women who fulfilled the inclusion criteria of puerperal sepsis were included and data from their files were collected and entered in SPSS version 19.0. Mean and standard deviations were calculated for continuous variables while for categorical variable proportion and percentages were used.

**Results:**

This retrospective record review in five years showed the 396 patients had P-sepsis, among them 44 patients had severe sepsis with organ dysfunction. The culture was positive in 26(59%) with trend of E-coli in 9(20%) Among them 12(27%) had serum lactate more than ≥4mmol/L. Central venous pressure monitoring with fluid resuscitation was done as per protocol of survival bundle given to all 12(100%), Vasopressin was needed in half of these patients 6(50%). Amid 44 patients of severe sepsis 29(66%) were admitted to special care, while 15(34%) required intensive care admissions. Our 7(16%) patients failed to survive. All of them had multi-organ failure.

**Conclusion:**

There was moderate adherence of modified surviving sepsis campaign resuscitation bundles. Further improvement in compliance is warranted.

## INTRODUCTION

Millennium development goal (MDG) five was to reduce maternal mortality from background of 1990, by 75% by 2015. There was much improvement by 50% reduction however, it was not up to the mark.^1^ The strategic development goal(SDG) targeted to reduce maternal mortality to<70/100,000.^2^ Pakistan reports showed the maternal mortality is 178/100,000, this is far behind then the target.^1^ A study in 2014 reported as hemorrhage leads to 27% while sepsis causes 11% of maternal deaths.^3^ Similarly a systematic review also reported, sepsis as the direct maternal mortality in Asia by 11.6% while in developed countries it is only 2.1%.^4^

The exact burden of sepsis is difficult to gauge due to differences in definition. In 2019 definition of sepsis and septic shock has been revised at the third international consensus sepsis definition (Sepsis-3).^5^ There is a lot of literature which highlighted the risk factors associated with sepsis like un-booked, multiparity, anemia, caesarean section and so on and forth.^6^ However still we fail to achieve any improvement. Dellinger et al.’s study reported that puerperal pyrexia and sepsis are highly preventable problems occurring among the leading causes of maternal morbidity and mortality in both developing and developed countries.^7^ The issuance of international guidelines for management of severe sepsis and septic shock called as “Modified surviving sepsis campaign resuscitation bundles in puerperal sepsis” in 2008^8^, followed by Royal College of obstetricians and gynecologists in 2012, released early warning signs for the diagnosis criteria for ICU admission. It strongly recommends that first three hours after presentation are very crucial. During these hours certain tasks are predefined, this includes initiation of broad-spectrum antibiotics within one hour after obtaining blood and other relevant cultures, measuring serum lactate. In case of serum lactate being higher than 4mmol/l or hypotension, crystalloid should be started with the rate of 30ml/kg in order to achieve central venous pressure of ≥ 8mmHg and mean arterial pressure ≥65mm Hg. Vasopressors should also be considered, if patient is not responding to fluid resuscitation. Central venous oxygen saturation should also be maintained at ≥70%.^9^,^10^

Pakistan has a high mortality due to puerperal sepsis. We are not sure that our patients with Puerperal sepsis are properly managed in light of international guidelines. There is no study published so far that focus on these lines of management in our region. Hence this study will assess the factors contributing to puerperal sepsis and compliance of “Modified surviving sepsis campaign resuscitation bundles in puerperal sepsis” for the management of puerperal sepsis. Furthermore, it also measures puerperal sepsis related maternal mortality and morbidity at Aga Khan university Hospital. The results of this study and recommendations in the light of this data will help in early diagnoses and treatment of cases with puerperal sepsis. We anticipate that this study will improve awareness in caregivers along with its implementation makes revolutionary improvement in maternal morbidity and mortality due to puerperal sepsis.

## METHODS

This was a retrospective record review for five years from January 2011 to December 2015. All women who were admitted with suspicion of puerperal sepsis during pregnancy till 42 days after delivery were recruited and data from their files were collected. Women who were immunocompromised due to any other prior illness or using immunosuppressant were excluded. Puerperal sepsis is defined as the infection of the genital tract occurring at labor or within 42 days of the postpartum period. Among these patients who have had sepsis-induced organ dysfunction or tissue hypoperfusion were labelled as Severe sepsis. Septic shock is defined as the persistence of hypoperfusion despite adequate fluid replacement therapy along with constant severe abdominal pain and tenderness unrelieved by usual analgesia.

All patients who developed sepsis during pregnancy, labor or within 42 days after delivery were included in this study. Their files were reviewed. Details like age, gestational age, and mode of delivery, use of antibiotics, culture results, fluid therapy and serum lactate level were retrieved. Their course of hospital stays and management from the first encounter in emergency room were taken along with the details of time about what, when, who and how the management of P-sepsis was done, were recorded from the files

### Data management and analysis:

Data was entered and analyzed in Statistical Package for Social Sciences (SPSS) version 19.0. Mean and standard deviations were calculated for continuous variables such as maternal age, gestational age at delivery. Clinical Presentations, culture results and compliance of sepsis surviving bundle were calculated in percentage. Permission from ethical review board of the institution was taken with reference number (3528-OBS-ERC-15). The information was taken from medical records remained confidential. Patient name and identity were not disclosed at any time.

## RESULTS

There were 396 patients who presented to emergency with suspicion of puerperal sepsis. The mean age of patients with P-sepsis was 28.51(SD±4.75). The most prevalent contributing factor was anemia in 250(63%) followed by diabetes in 77(19%) while preterm pre-labor rupture of membrane in 62(16%) of patients. Two third of patient delivered by Caesarean section. The common presentations were fever and tachycardia found in about 255(64%) of patients shown in Table-I.

**Table I:** Clinical factors associated with P-sepsis.

	Total N 396(%)
Anemia	250(63.1)
Diabetes	77(19.4)
Vaginal discharge + Pelvic infection	21(5.3)
PPROM	62(15.7)
***Mode of delivery***	
Vaginal	150(37.87)
Caesarean section	246(62.12)
Fever	257(64.9)
Rigors	187(47.2)
Tachycardia	254(64.1)
GIT symptoms	34 (8.58)
Lethargy	103(26)
Breast abscess	6(1.5)
UTI	14(3.5)
RTI	19(4.8)
Abdominal distention	32(8.08)
Offensive vaginal discharge	10(2.5)
Sub involution of uterus	3(0.8)
Maternal Mortality due to
severe sepsis	7(15.90)*
***Level of Care***	
Special Care	29(65.90)*
Intensive care unit	15(34.09)*

* N 44(Denominator).

Severe sepsis with organ dysfunction cases are shown in Table-II. There were 44(11%) of severe sepsis out of 396 patients. Further break up of organ failure revealed that liver was affected in 26(59%) followed by renal dysfuction 7(16%). Multi-organ involvement seen in 9(20%) of patients with severe sepsis. The culture was positive in 26(59%) with trend of *E-coli* in 9(20%) and Group-A streptococcus (GAS)in 5(11%).

**Table II:** Sepsis associated with organ dysfunction and microorganism.

Organ dysfunction	44(11.1)
Liver	26(59.09)
Renal	7(15.90)
Lungs	2(4.54)
Multi organ failure	9(20.45)
Organism Overall Culture positive	26(59.09)
GAS	5(11.36)
E-coli	9(20.45)
Staphylococcus	2(4.54)
Klebsella	3(6.81)
Pseudomonas	3(6.81)
MRSA	1(2.27)
Acitenobacter	3(6.81)

Among 396 patients with sepsis the adherence to survival bundle of P-sepsis depicted in Table-III. Overall, the culture was sent in half of the patients. Biomarkers like C-reactive protein was done in 25(6.3%) while serum lactate was measured in 19(5%) out of which 12 patients had the level ≥4mmol/L. Antibiotics were administered to 90(23%) within one hour of arrival. Sepsis with organ dysfunction was found in 44 patients, all patients were attended by consultant within three hours, among them 12(27%) had serum lactate more than ≥4mmol/L. Central venous pressure monitoring with fluid resuscitation was done as per protocol of survival bundle given to all 12(100%) patients with high serum lactate, while vasopressin was needed in half of these patients 6(50%).

**Table III:** Compliance of Survival Bundle in p-sepsis.

	Total N 396(%)
Obtain cultures	191(48.1)
Blood	145(36.6)
Urine	154(38.9)
High Vaginal swab	97(24.5)
Wound swab	10(2.5)
Fluid tap	8(2.0)
Measure serum lactate/CRP	44 (11.1%)
Administer Antibiotics (one hour)	90(22.7)
Severe Sepsis	Total N 44(%)
Consultant arrival in 3 hours	49(113.36)
CVO2	37(84.1)
Serum lactate ≥ 4	12(27.2)
Fluid resuscitation	12/12 (100)*
CVP	12 (100)*
Vasopressin	6(50)*

* Denominator is 12 who had S/lactate≥ 4mmol/L.

Management of sepsis with trends to antibiotics and further intervention required to control sepsis is shown in Table-IV. Amid 44 patients of severe sepsis 29(66%) were admitted to special care, while 15(34%) required intensive care admissions. Our 7(16%) patients expired. All of them had multi-organ failure.

**Table IV:** Medical and surgical management of P-sepsis.

Antibiotics	Total N 396(%)
Cephalosporin and metronidazole	275(69.4)
Co-amoxicillin	101(25.5)
clindamycin	3(8)
Imepenum	16(4)
Piperacillin	37(9.3)
Vancomycin	12(3.0)
Tecoplasmin	1(0.3)
polymyxin	2(0.5)
Surgical management Source Control (severe sepsis) *	N 44(Denominator)
Evacuation of uterus	2(4.54)
TOP/LSCS	10 (22.72)
Laparotomy	5(11.36)
Hystrectomy	2(4.54)
Resuturing	5(11.36)
Ultrasound guided aspiration	5(11.36)

* N 44(Denominator).

## DISCUSSION

This retrospective record review in five years showed the 396 patients had P-sepsis, among them 44(11.1%) patients had severe sepsis with organ dysfunction**.** The studies in Pakistan showed the rate between 3.9-6.3%,^11^,^12^ with maternal mortality related to P-Sepsis was between 13-20%.^13^-^15^ The available data in Pakistan is limited mainly hospital based data, the criteria of sepsis in also not clear.^16^

This study showed that anemia was found in 63% of patients with sepsis, while other studies conducted in a public hospital showed, almost all suffering from anemia and one third of women hemoglobin was less 5g/dl with sepsis.^11^,^12^ Our data set, catered only to patients with sepsis and showed that 62% had delivered by C-section, hence unable to check the rate of C-section in non-sepsis patients. An American study also exhibit the higher rate of C-section in sepsis patient with odd ratio of 1.58.^17^

Our study depict E-coli is the commonest organism followed by GAS. This has been further endorsed by a study in Zimbabwe, however confidential enquiries on maternal mortality UK suffice Group A streptococcus as the major culprit of p-sepsis related mortality.^9^,^18^

The compliance of Sepsis survival bundle was variable. The cultures were obtained in half of the patients with sepsis. Similarly, other components were also accomplished in severe sepsis, but not in all patients with sepsis. Multi organ failure leads to most of the death 9(78%) in our study, this was further endorsed by a local study, showed that in majority of maternal mortality is associated with Multi organ failure.^19^ Therefore, strict compliance to sepsis survival bundle could improve maternal morbidity and mortality. World Health Organization Global Sepsis Study (GLOSS), shows the death rate due to sepsis in low middle income(LMIC) and low income (LIC) countries range from 7-14.8% respectively, this far more then upper middle income countries(UMIC) i.e. 1.1%.^20^ It is a strong recommendation to take culture and start antibiotics within one hour of arrival. Therefore, broad spectrum antibiotics usually starts within one hour after collecting relevant cultures.^7^

The most common antibiotics used in this study was the combination of cephalosporin and metronidazole followed by Co-amoxiclav. However, this was the choice up to 2005, but due to lack of coverage against Methicillin-resistant staphylococcus aureus (MRSA), Extended Spectrum Beta-Lactamase (ESBL) and pseudomonas along with increased risk of C.difficile infection. Piperacillin/ tazobactam or carbapenem with clindamycin is the first choice,^10^,^21^ On the other hand these empirical antibiotics could develop multi drug resistance.^22^ Molecular technology has been evolving to receive culture results in < one hour.^23^ It has been proven from the research conducted in US health system that resuscitation survival bundle is directly correlated with the duration of compliance, by reduction in mortality by 7% in consequent quarters. Those hospitals who used this for more than three years showed reduced mortality by 36%.^24^ If the time limits to three hours, further improved the survival in cost effective manner.^25^ An update in 2018 by Levy suggests, more aggressive management by one hour.^26^ However adopting to one hour bundle is a concern, leads to more pressure on emergency department with over treatment of patients among sepsis without shock.^22^ Procalcitonin and C-Reactive proteins are non-specific markers. The use of broad-spectrum antibiotics empirically leads to cross resistance. The availability of specific devices that identify the causative organism and sensitivity to antibiotics in one hour is warranted.^23^

LMIC countries like Pakistan, being an under-resourced and low resilience health system with inadequate staffing, low funds, lack of expertise, substandard care, absence of national guidelines and weak referral system has negative impact on the management of sepsis. Similarly, inadequate surgical and critical care capacity, misuse of antibiotics and increasing Anti-microbial resistance further worsen the situation.^20^ The other factors that increases the risk of sepsis are illiteracy, poverty, poor sanitation, delivery at home, inadequate transport facilities, limited resources and unequipped health facilities with limited staffing and suboptimal care.

### Limitations of the study:

It is retrospective record review with single hospital-based data. This is one of the private university hospitals mainly catered one socioeconomic class, so the incidence was found lower than the government hospital.

## CONCLUSION

Anemia and operative deliveries are the major risk factors in sepsis. There was moderate adherence of modified surviving sepsis campaign resuscitation bundles. Further improvement in compliance warranted.

### Authors’ Contribution:

**FY** conceived the idea, develop study protocol, responsible for accuracy, integrity of the data and prepared the manuscript.

**AM** helped in acquisition of data, identify the key parameters to assess. She was also responsible for accuracy and integrity of the data**.**

**AS** helped in Data acquisition &manuscript editing.

**SS** helped in proposal writing, statistical analysis and interpretation of results
